# 5-(5-Aryl-1,3,4-oxadiazole-2-carbonyl)furan-3-carboxylate and New Cyclic *C*-Glycoside Analogues from Carbohydrate Precursors with MAO-B, Antimicrobial and Antifungal Activities

**DOI:** 10.3390/molecules17067010

**Published:** 2012-06-07

**Authors:** Mohamed Mohamed El-Sadek, Seham Yassen Hassan, Nagwa Said Abd El-Dayem, Galila Ahmed Yacout

**Affiliations:** 1 Chemistry Department, Faculty of Science, Alexandria University, Alexandria 21231, Egypt; Email: sehamyassen@yahoo.com (S.Y.H.); nagwa_abdeldayem@yahoo.com (N.S.A.E.-D.); 2 Biochemistry Department, Faculty of Science, Alexandria University, Alexandria 21231, Egypt; Email: galila_69@yahoo.com

**Keywords:** carbohydrazone, isopropylidene, triazole, oxadiazole, monoamine oxidase-B

## Abstract

Cyclization of acyclic *C*-glycoside derivatives **1a,b** to **2a,b** as the major isomers, and **4a,b** as the minor isomers were carried out. The isopropylidene derivatives **3a,b** were prepared, as well as the hydrazide derivative **6**, which was condensed with a variety of aldehydes to give hydrazones **7a–e** which were also prepared from the compounds **12a–e**. Acetylation of **7a,d** gave the corresponding acetyl derivatives **8a,d**, respectively. In addition, the dicarbonyl compound **9** was prepared in the hydrate form, which reacted with a number of aroylhydrazines to give the corresponding bisaroyl-hydrazones **10a–d**, which were cyclized into 1,3,4-oxadiazoles **11a–d**. Furthermore, two of the prepared compounds were examined to show the ability to activate MAO-B. In addition a number of prepared compounds showed antibacterial and antiviral activities.

## 1. Introduction

*C*-glycosides have received a great deal of attention from the synthesis and medicinal chemistry community, due to their increased stability to hydrolysis as well as their presence in a number of interesting natural products [1]. Furthermore, heterocyclic compounds containing the five-membered oxadiazole nucleus possess a diversity of useful biological effects. Substituted 1,3,4-oxadiazoles are of considerable pharmaceutical interest, for instance, 2-amino-1,3,4-oxadiazoles act as muscle relaxants [2] and show antimitotic activity. Anti-inflammatory [3,4], antimicrobial [4], anti-hepatitis B [5] and anti-diarrheal activity [6] of some new 1,3,4-oxadiazole derivatives was also reported. Recently several 1,3,4-oxadiazole derivatives were identified as potentially active antimycobacterial [7,8], antitubercular [9], anticonvulsant [10] and anticancer [11] agents, and also reported as enzyme tyrosinase inhibitors [12]. In light of these interesting biological activities, it became interested in synthesizing some new *C*-glycosides of substituted 1,3,4-oxadiazole derivatives and evaluating their antimicrobial potential.

## 2. Results and Discussion

### 2.1. Chemistry

It has been shown [13] that the acid-catalysed, intramolecular dehydration of **1b** using conc. HCl (0 °C) yields a mixture of a major D-*ribo* anhydro isomer **2b** (with inversion of configuration), and a minor D-*arabino*-anhydro isomer **4b** (with retention of configuration). On the other hand, the dehydration of **1a** with aqueous acetic acid (10%) under reflux [14], afforded a mixture of anhydro derivatives **2a** (major isomer) and **4a** (the minor one) and in addition, a mixture of **2b** and **4b** were obtained from **1b** using aqueous acetic acid (10%). The minor isomer **4a** could be isolated as a solid.

**Scheme 1:**
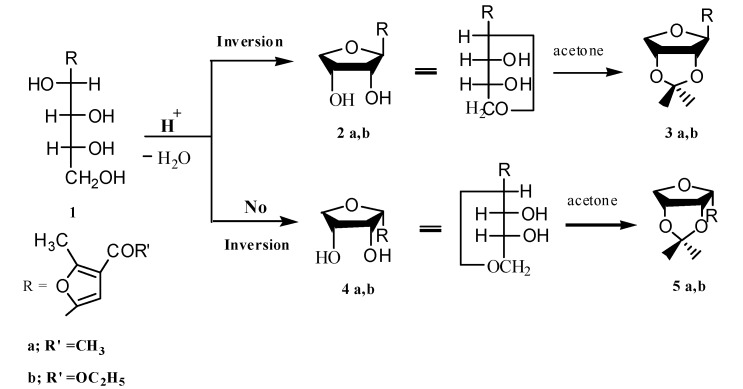
Synthesis of isopropylidene derivatives **3a,b**.

The anomeric configurations [15] of these anhydro derivatives were ascertained from the ^1^H-NMR spectra of their isopropylidene derivatives **3a,b** which were prepared by an improved procedure [16] through treatment of the crude anhydro derivatives with acetone in the presence of catalytic amount of *p*-toluenesulphonic acid (see Experimental and Scheme 1).

**Figure 1:**
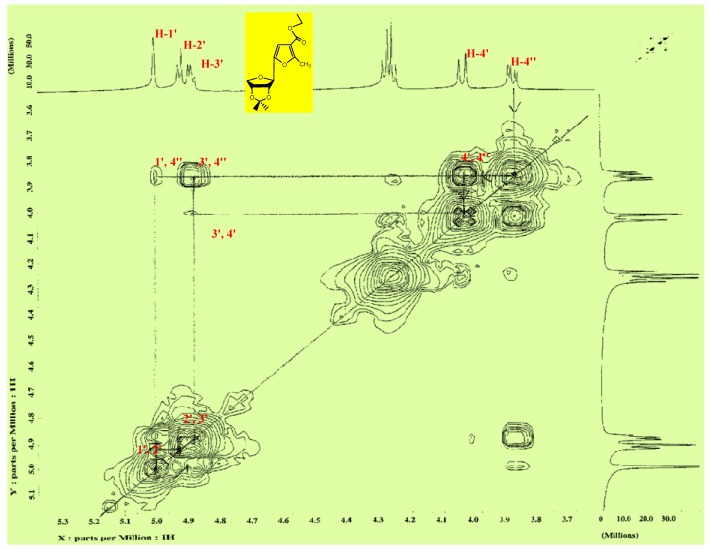
2D ^1^H-NMR spectrum of compound **3b**.

**Scheme 2:**
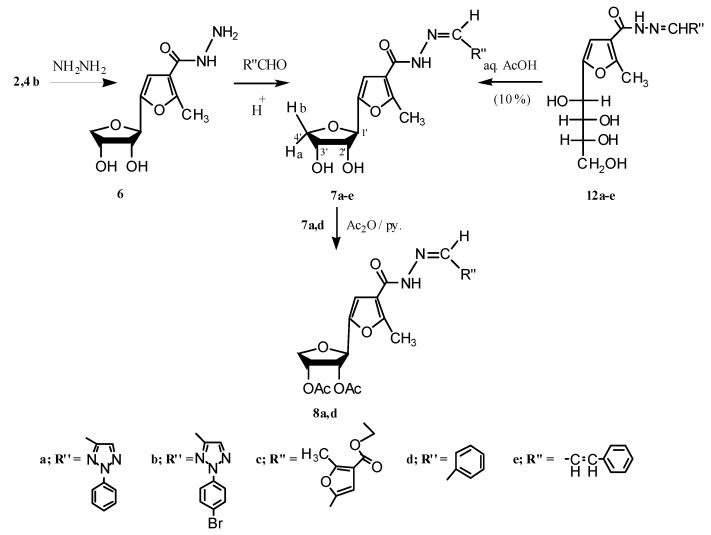
Synthesis of carbohydrazone derivatives.

^1^H-NMR spectra (CDCl_3_) of compounds **3a,b** displayed the two methyl protons signals of the 2,2-dimethyldioxolane ring at δ (1.544–1.352) having Δδ 0.187. The signals of the sugar protons of these isopropylidene derivatives were assigned from the 2D ^1^H-NMR spectrum of compound **3b** (Figure 1), and the characteristic chemical shifts as compared with those reported for anhydro analogues [16], whereby the C-1' proton appears as a singlet at δ 5.00 (*J*_1',2'_ = 0.00 Hz). Confirmation of the anomeric configuration of the isopropylidene derivatives **3a,b** can be obtained from the zero coupling constant value (*J*_1',2'_ = 0.00 Hz), which is an unequivocal [16,17] proof for the *trans* arrangement of the H-1' and H-2' (β-D-configuration) as well as from the Δδ value (0.187) [15,18,19]. Boiling of the crude **2b,4b** mixture with hydrazine hydrate resulted in the formation of **6**, which upon condensation with a number of aldehydes afforded the corresponding anhydrohydrazone derivatives **7a–e** which were also obtained as only one isomer (inversion of configuration at C-1') by boiling the compounds **12a–e** [20,21] with aqueous acetic acid under reflux. The assignment of the signals for the sugar protons in the ^1^H-NMR spectra of compounds **7a–e** were based on the 2D ^1^H-NMR spectrum of compound **7e**. In addition, acetylation of the anhydrohydrazones **7a,d** afforded the corresponding *O-*acetyl derivatives **8a,d**, respectively (see Experimental and Scheme 2).

**Scheme 3:**
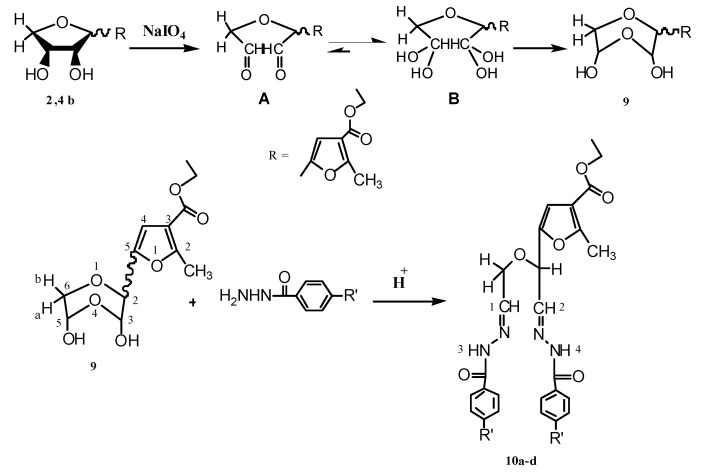
Synthesis of bisaroylhydrazone derivatives **10a–d**.

**Scheme 4:**
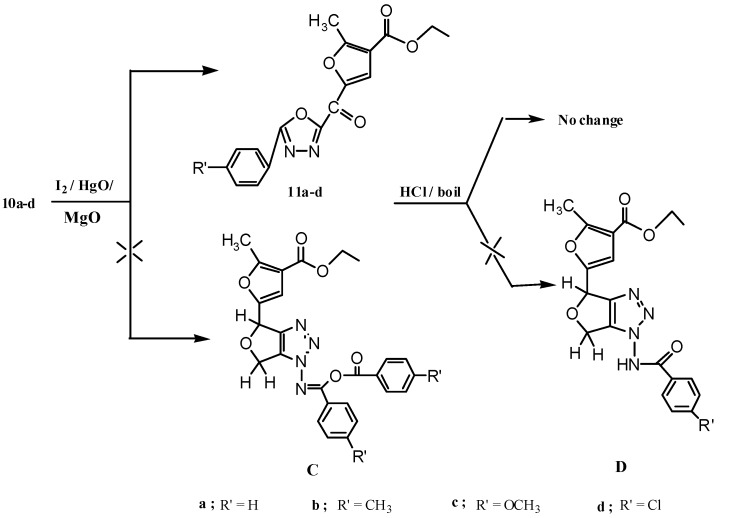
Synthesis of 1,3,4-oxadiazole derivatives **11a–d**.

Periodate oxidation [22] of the prepared anhydro-derivatives **2b,4b**, gave the corresponding dialdehyde in the hemialdal structure **9** [22,23]. Furthermore, condensation of dialdehyde **9** with two molar equivalents of aroylhydrazines, afforded the corresponding bisaroylhydrazones **10a–d**. ^1^H-NMR spectra of compounds **10a–d** (DMSO-d_6_), showed the two (NH) protons at δ (11.78–11.55) as two singlets, followed by the aromatic protons as a multiplet at δ (7.82–7.00), two (CH=N) as a doublet at 7.9 for H_(2)_ and a multiplet at 7.7 for H_(1)_, and the proton at position-4 in the furan ring as a singlet at 6.7 ppm. The methine proton was shown as a doublet at δ 5.1, followed by a multiplet at 4.1 ppm for the protons of the two methylene groups. Oxidative cyclization of the prepared bisaroylhydrazones **10a–d** and a physical and chemical study of the oxidation products, revealed that their properties could not be reconciled with that of 1,2,3-triazole derivatives C [24,25,26,27] but rather was compatible with that of 1,3,4-oxadiazole derivatives **11a–d**, that were obtained in appropriate yields. Infrared spectra of these compounds showed no enol benzoate group as expected for the 1,2,3-triazole derivatives C, and showed instead a band at 1,667–1,660 cm^−1^, which was attributed to the conjugated carbonyl group of compounds **11a–d** (see Experimental and Scheme 3 and Scheme 4).

**Scheme 5:**
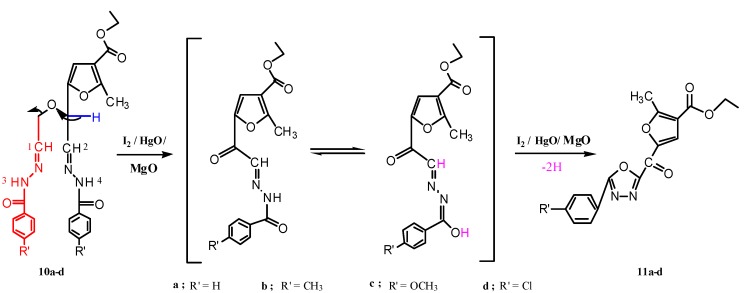
Proposed mechanism for formation of ethyl 2-methyl-5-(5-aryl-1,3,4-oxadiazole-2-carbonyl)furan-3-carboxylate.

Furthermore, ^1^H-NMR spectra (CDCl_3_) of these products showed the disappearance of signals corresponding to two (NH) and two (CH=N), methine and methylene protons. Indeed, it is noteworthy that the integration of the aromatic part (δ 8.22–7.36 ppm), referred to one aromatic ring only, in accord with structures **11a,b**,**d**. In addtion, these oxidative cyclization products displayed the proton at position-4 in the furan ring as the most downfield signal at δ (8.49–8.48) ppm; this proton resonated at a lower field region than that expected in CDCl_3_, which may be attributed to the electron withdrawing effect of the carbonyl group on the neighboring carbon atom, as well as conjugation with the phenyl oxadiazole moiety. In addition, the structure of the oxidation products **11a–d** was further supported through their boiling with hydrochloric acid, which didn’t afford the corresponding amine derivatives D as expected from 1,2,3-triazole derivatives (see Experimental part and Scheme 4). Moreover, the proposed mechanism for formation of **11a–d** may proceed via elimination of one aroylhydrazone part (oxidation in presence of iodine and mercuric oxide) due to the bulkiness of the molecule, followed by oxidative cyclization of the other aroylhydrazone part to afford compounds **11a–d** (Scheme 5).

### 2.2. Pharmacological Screening

#### 2.2.1. MAO-B Activity

##### 2.2.1.1. Effect of Tested Compounds on MAO-B

This study aimed to evaluate the effect of two selected newly prepared compounds **7c,e** on MAO-B activity given the biological importance of MAO-B [28,29,30,31,32,33,34,35,36,37,38].

##### 2.2.1.2. Determination of V_max_ and _Km_

**Figure 2:**
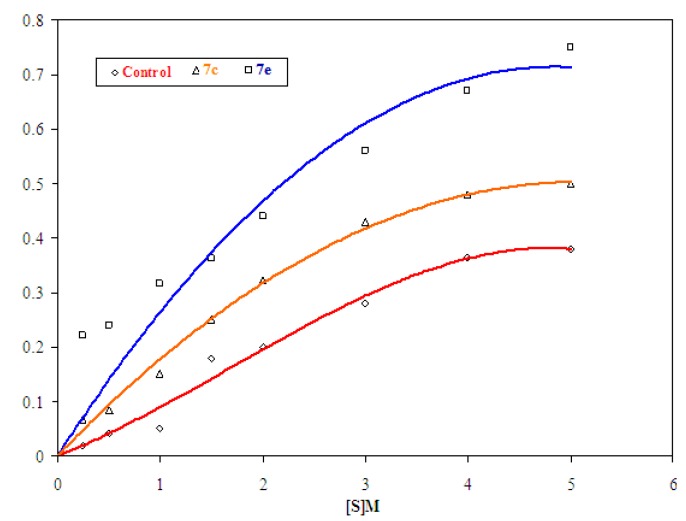
Effect of substrate concentration on the rate of MAO-B catalyzed reactions in the presence of the examined compounds **7c,e** compared to control.

TheV_max_ and K_m_ of the MAO-B catalyzed reaction in the presence or absence of each examined compound was carried out by plotting V against [S], each separately. The obtained results revealed that MAO-B was activated in the presence of compounds **7c** and **7e**, each separately, by 6.57- and 6.97-fold, respectively. In addition, the MAO-B catalyzed reactions in the presence of compounds **7c** and **7e** have V_max_ equal to 0.49 and 0.71, respectively. Meanwhile the K_m_ values were 1.72 and 1.32, respectively. Our obtained data showed that compound **7e** was an effective MAO-B activator, which increases the affinity of substrate to bind with the active site of MAO-B enzyme. That may be attributed to the presence of highly conjugated system with the cinamyl group in **7e** as compared with bulky furan ring in **7c** (Table 1, Figure 2).

**Table 1:** Effect of substrate concentration on the rate of MAO-B catalyzed reactions in presence of the examined compounds **7c** and **7e**, compared to control.

Substrate conc.	Rate		
	Control	7c	7e
0.25 × 10^−3^	0.020	0.068	0.222
0.50 × 10^−3^	0.042	0.085	0.240
1.00 × 10^−3^	0.050	0.151	0.315
1.50 × 10^−3^	0.180	0.250	0.363
2.00 × 10^−3^	0.200	0.322	0.440
3.00 × 10^−3^	0.280	0.430	0.560
4.00 × 10^−3^	0.365	0.481	0.674
5.00 × 10^−3^	0.380	0.498	0.700

#### 2.2.2. Antibacterial and Antifungal Activities

The compounds **3b**, **6**, **7c,e**, **10a–c** and **11a,b,d** have been studied for their antibacterial and antifungal activities against four bacterial species (*Escherichia coli*, *Bacillus* sp., *Staphylococcus* sp., and *Sarcina* sp.) and six fungal species (*Aspergillus niger*, *Aspergillus fmigatus*, *Alternaria* sp., *Fusarium* sp., *Chaetomium* sp., and *Penicillium* sp.) using the Nutrient Agar (NA) and Sabouraud Dextrose Agar (SDA) diffusion methods, respectively, in DMSO solvent (Table 2).

**Table 2:** Inhibition zones of tested compounds against selected microorganisms (mm).

**Compound No.**	**Bacteria**					
	**Gram positive**			**Gram negative**		
	***Bacillus* sp.**	***Sarcina* sp.**	***Staphylococcus* sp.**	***E. coli***		
**3b**	….	….	….	….		
**6**	….	….	2	….		
**7c**	….	….	0.5	….		
**7e**	….	….	2	….		
**10a**	….	….	….	….		
**10b**	….	….	….	….		
**10c**	….	….	….	….		
**11a**	….	….	1	….		
**11b**	….	….	….	….		
**11d**	….	….	….	….		
**Compound No.**	**Fungi**					
	***Aspergillus niger***	***Aspergillus fumigatus***	***Alternaria*** **sp.**	***Fusarium * sp.**	***Chaetomium * sp.**	***Penicilliu * sp.**
**3b**	1	1	….	2	….	….
**6**	….	….	1	1	2	2
**7c**	1	1	0.5	2	….	0.5
**7e**	….	2.5	0.5	2	….	1
**10a**	….	….	….	….	….	….
**10b**	1	….	….	….	….	….
**10c**	1	….	….	….	….	….
**11a**	0.5	….	0.5	….	….	2
**11b**	….	….	….	….	….	….
**11d**	….	….	….	2	….	….

….: No effect.

It was found that they were active against Gram positive bacteria (*Staphylococcus* sp.). In addition, the replacement of the bulky furan ring in compound **7c** by the cinamyl group in compound **7e**, increased the antibacterial activity against Gram positive bacteria (*Staphylococcus* sp.), due to the conjugation caused by the cinamyl group [39]. With respect to antifungal activity, the compounds **3b**, **6**, **7c,e**, and **11a** were found to be generally active against the tested fungi.

## 3. Experimental

### 3.1. Chemistry

Melting points were determined with a Melt-temp. apparatus and are uncorrected. TLC was performed on Baker-Flex silica gel 1B-F plates and the spots were detected by UV light absorption. IR spectra were recorded on Perkin Elmer. USA Spectrometer. ^1^H-NMR, ^13^C-NMR, and 2D ^1^H-NMR were recorded on a JEOL JNM ECA 500 MHz instrument using tetramethylsilane as an internal standard. Mass spectra were recorded on GCMS solution DI Analysis Shimadzu Qp-2010 Plus. Optical rotation was obtained at 22 °C with a Perkin-Elmer Model 241 Polarimeter 10 cm, 1 mL microcell. Microanalyses were performed at the faculty of Science, Cairo University, Cairo, Egypt. Solutions were evaporated under diminished pressure unless otherwise stated. The ChemDraw-Ultra-8.0 has been used in generating the nomenclature of the prepared compounds.

#### 3.1.1. *Ethyl 5-(2',3'-Dihydroxytetrahydrofuran-1'-yl)-2-methylfuran-3-carboxylates* **2b,4b**

Compound **1b** (15.0 g, 54.74 mmol) was boiled under reflux with aqueous acetic acid (300 mL, 10%) for 5 h, while the reaction was monitored by TLC (chloroform-methanol, 20:1, V/V), the starting material disappeared and a more mobile spot (Rf: 0.5) was obtained. The solvent was evaporated under diminished pressure and washed with toluene (3 times, 10 mL each) to obtain **2b** as a yellow thick syrup along with a solid mass of **4b**. Separation of **4b** from the syrup was carried out by washing the mixture with ethanol where it was partially soluble in ethanol on cold; the overall yield was 69.7%; the solid mass was recrystallized from ethanol as colourless needles; IR (KBr): 3,426 (OH), 1,712 cm^−1^ (CO-ester).

#### 3.1.2. Synthesis of Isopropylidene Derivatives

General Methods. A solution of **2a,4a**[22] or **2b,4b** mixture (4.42 mmol) in dry acetone (100 mL) was treated with *p*-toluenesulphonic acid (10.98 mmol) and stirred at room temperature. The reaction mixture was monitored by TLC (hexane-ethyl acetate, 7:1, V/V), the starting material disappeared after 7 h and a more mobile spot appeared. The mixture was then poured onto a saturated solution of sodium bicarbonate, extracted with chloroform, the organic layer washed with water and dried over anhydrous sodium sulphate. Evaporation of the dried filtrate gave pale yellow needles.

*1-[5-(2',3'-O-Isopropylidene-β-D-erythrofuranosyl)-2-methylfuran-3-yl]ethanone* (**3a**). Yield 51%; recrystallized from ethanol as colourless needles, mp 97–99 °C; R_f_: 0.35 (hexane-ethyl acetate, 7:1, V/V), IR (KBr): 1,682 (CO-acetyl), 1,604, 1,571 cm^−1^ (C=C); ^1^H-NMR (CDCl_3_); δ: 1.358, 1.544 (2s, 6H, CMe_2_, Δδ 0.187), 2.376 (s, 3H, COCH_3_), 2.559 (s, 3H, CH_3(furan)_), 3.909 (dd, 1H, H-4'a, *J*_3',4'a_ = 3.85 Hz, *J*_4'b,4'a_ = 10.70 Hz), 4.049 (d, 1H, H-4'b, *J*_4'b,4'a_ = 10.70 Hz), 4.892–4.937 (m, 2H, H-2', H-3'), 5.001 (s, 1H, H-1'), 6.475 (s, 1H, CH_furan_); MS: *m/z* (%), 267 (3.50, M^+^+1), 266 (4.82, M^+^), 137 (36.26), 125 (21.36), 111 (35.32), 109 (21.18), 99 (21.56), 97 (52.51), 95 (33.70), 85 (46.86), 83 (49.75), 81 (32.98), 73 (26.34), 71 (66.24), 69 (61.30), 67 (23.40), 60 (25.40), 57 (100), 56 (21.33), 55 (65.29).

*Ethyl 5-(2',3'-O-isopropylidene-β-D-erythrofuranosyl)-2-methylfuran-3-carboxylate* (**3b**). Yield (90%); recrystallized from ethanol as colourless needles, mp 70–72 °C; R_f_: 0.63 (hexane-ethyl acetate, 7:1, V/V); [a]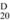 −69.03; IR (KBr): 1,709 (CO-ester) 1,613, 1,583 cm^−1^ (C=C); ^1^H-NMR (CDCl_3_); δ: 1.324 (t, 3H, CH_3(ester)_, *J* = 6.90 Hz), 1.352, 1.538 (2s, 6H, CMe_2_, Δδ 0.187), 2.542 (s, 3H, CH_3(furan)_), 3.870 (dd, 1H, H-4'a, *J*_3',4'a_ = 3.80 Hz, *J*_4'b,4'a_ = 10.70 Hz), 4.029 (d, 1H, H-4'b, *J*_4'b,4'a_ = 10.70 Hz), 4.260 (q, 2H, CH_2(ester)_, *J* = 6.90 Hz), 4.876–4.928 (m, 2H, H-2', H-3'), 5.003 (s, 1H, H-1'), 6.486 (s, 1H, CH_furan_); MS: *m/z* (%), 297 (6.3, M^+^+1), 296 (20.6, M^+^), 182 (100), 181 (30.6), 145 (60), 153 (43), 137 (25), 136 (25.6), 105 (21.3), 80 (27.5), 79 (28.8), 69 (38.1), 65 (28.8), 60 (13.8), 59 (76.3), 56 (25), 55 (30.6), 53 (33.8), 52 (44.4), 51 (60), 50 (25).

*5-(2',3'-Dihydroxytetrahydrofuran-1'-yl)-2-methylfuran-3-carbohydrazide* (**6**). A solution of **2b,4b** (2.0 g, 7.81 mmol) in ethanol (10 mL) was treated with hydrazine hydrate (4 mL) under reflux for one hour, while the reaction was monitored by TLC (chloroform-methanol, 6:1, V/V) which revealed the absence of the starting material and formation of two more mobile spots, R_f_: 0.49 (major) and 0.74 (minor). After cooling **6** separated out, was filtered off, washed with a little ethanol, and dried; yield 65%. It was recrystallized from ethanol as colourless needles, mp 185–187 °C. TLC showed one spot only, R_f_: 0.49 (chloroform-methanol, 6:1, V/V); The minor isomer was left in the mother liquor; [a]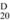 −58.98; IR (KBr): 3,384, 3,314 (OH and NH_2_), 3272 (NH), 1,636 cm^−1^ (CO-amide); ^1^H-NMR (DMSO-d_6_); δ: 2.463 (s, 3H, CH_3(furan)_), 3.570 (dd, 1H, H-4'b, *J*_3',4'b_ = 2.30 Hz, *J*_4'b,4'a_ = 9.15 Hz), 3.979 (dd, 1H, H-4'a, *J*_3',4'a_ = 4.60 Hz, *J*_4'b,4'a_ = 9.15 Hz), 4.029–4.066 (m, 2H, H-3', H-2'), 4.312 (s, 2H, NH_2_), 4.395 (d, 1H, H-1', *J*_1',2'_ = 6.90 Hz), 4.985 (d, 1H, 2'-OH, *J*_2',OH_ = 4.60 Hz), 5.076 (d, 1H, 3'-OH, *J*_3',OH_ = 6.85 Hz), 6.715 (s, 1H, CH_furan_), 9.243 (s, 1H, NH). After shaking with D_2_O, the NH, NH_2_, and the two hydroxyl protons disappeared; MS: *m/z* (%), 243 (2.19, M^+^+1), 242 (16.89, M^+^), 224 (0.47, M^+^-H_2_O), 212 (11.54, M^+^-2NH), 211 (100, M^+^-NHNH_2_); Anal. Calcd for C_10_H_14_N_2_O_5_: C, 49.58; H, 5.84; N, 1.55%; found: C, 49.58; H, 5.83; N, 1.56%.

#### 3.1.3. Reactions of **6** with a Number of Aldehydes

##### General Methods

Method A. A solution of **6** (0.5 g, 2.066 mmol) in ethanol (5 mL) containing acetic acid (0.1 mL) was treated with the corresponding aldehyde (2.066 mmol), and the reaction mixture was refluxed on water bath for 10 min. After cooling the product 3-carbohydrazone derivative that separated out, was filtered off, washed with little ethanol, and dried.

Method B. A solution of (1',2',3',4'-tetrahydroxybutyl)furan-3-carbohydrazone **12a–e** [20,21] (4.799 mmol) was refluxed with aqueous acetic acid (10%) for 5 h. After cooling the compounds **7a–e** that separated out as a crystalline mass were filtered off, washed with little ethanol, and dried.

*5-(2',3'-Dihydroxytetrahydrofuran-1'-yl)-2-methyl-N-[(2-phenyl-2H-1,2,3-triazol-4-yl)methyl-ene]furan-3-carbohydrazone* (**7a**). Yield of method A 87%; recrystallized from ethanol as colourless needles, mp 209–210 °C; R_f_: 0.39 (chloroform-methanol, 15:1, V/V); [a]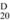 −37.59; IR (KBr): 3,395 (OH), 3,108 (NH), 1,669 (CO-amide), 1,638 cm^−1^ (C=N); ^1^H-NMR (DMSO-d_6_); δ: 2.524 (s, 3H, CH_3(furan)_), 3.616 (dd, 1H, H-4'b, *J*_3',4'b_ = 2.30 Hz, *J*_4'b,4'a_ = 9.20 Hz), 4.008–4.026 (m, 1H, H-4'a), 4.036–4.095 (m, 2H, H-3', H-2'), 4.493 (d, 1H, H-1', *J*_1',2'_ = 6.90 Hz), 5.043 (ss, 1H, 2'-OH), 5.142 (d, 1H, 3'-OH, *J*_3',OH_ = 6.15 Hz), 6.860 (s, 1H, CH_furan_), phenyl protons: 7.432 (t, 1H,*p*-H), 7.568 (t, 2H, *O*-H), 8.017 (d, 2H, *m*-H), 8.401 (s, 1H, CH=N), 8.537 (s, 1H, CH_triazole_), 11.610 (s, 1H, NH). After shaking with D_2_O, the NH proton and the two hydroxyl protons disappeared; MS: *m/z* (%), 399 (0.89, M^+^+2), 398 (5.36, M^+^+1), 397 (23.99, M^+^), 379 (3.55, M^+^-H_2_O), 324 (21.85), 253 (11.94, M^+^-phenyltriazole moiety), 211 (100), 137 (35.81), 77 (20.56, C_6_H_5_); Anal. Calcd for C_19_H_19_N_5_O_5_: C, 57.42; H, 4.80; N, 17.64%; found: C, 57.43; H, 4.82; N, 17.62%.

*N-[(2-(p-Bromophenyl)-2H-1,2,3-triazol-4-yl)methylene]-5-(2',3'-dihydroxytetrahydrofuran-1'-yl)-2-methylfuran-3-carbohydrazone* (**7b**). Yield of method A 84%; recrystallized from ethanol as colourless needles, mp 241–243 °C; R_f_: 0.38 (chloroform-methanol, 15:1, V/V); [a]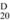 −31.39; IR (KBr): 3,423 (OH), 3,249 (NH), 1,653 (CO-amide), 1,618 cm^−1^ (C=N); ^1^H-NMR (DMSO-d_6_); δ: 2.519 (s, 3H, CH_3(furan)_), 3.617 (dd, 1H, H-4'b, *J*_3',4'b_ = 2.30 Hz, *J*_4'b,4'a_ = 9.95 Hz), 4.019 (dd, 1H, H-4'a, *J*_3',4'a_ = 3.85 Hz, *J*_4'b,4'a_ = 9.95 Hz), 4.080–4.109 (m, 2H, H-3', H-2'), 4.493 (d, 1H, H-1', *J*_1',2'_ = 6.90 Hz), 5.051 (ss, 1H, 2'-OH), 5.147 (d, 1H, 3'-OH, *J*_3',OH_ = 6.10 Hz), 6.857 (s, 1H, CH_furan_), *p*-bromophenyl protons: 7.752 (d, 2H, *m*-H), 7.954 (d, 2H, *o*-H), 8.422 (s, 1H, CH=N), 8.518 (s, 1H, CH_(triazole)_), 11.630 (s, 1H, NH). After shaking with D_2_O, the NH proton and the two hydroxyl protons disappeared; ^13^C-NMR; δ: 14.00 (C-13), 70.91 (C-4'), 73.32 (C-3'), 75.33 (C-2'), 76.50 (C-1'), 107.79 (C-12), 115.11 (C-11), 120.92 (C-10), 121.23 (C-9), 133.27 (C-8), 135.16 (C-7), 137.74 (C-6), 138.52 (C-5), 146.50 (C-4), 151.71 (C-3), 158.19 (C-2), 159.84 (C-1); MS: *m/z* (%), 478/476 (2.03, 2.17, M^+^+1), 477/475 (8.85, 9.10, M^+^), 459/457 (1.25, 1.24, M^+^-H_2_O), 253 (10.01, M^+^-*p*-bromophenyl triazole moiety), 211 (100), 137 (34); Anal. Calcd for C_19_H_18_BrN_5_O_5_: C, 47.90; H, 3.83; N, 14.71; Br, 16.77%; found: C, 47.91; H, 3.81; N, 14.70; Br, 16.78%.

*Ethyl 5-[(2-(5-(2',3'-dihydroxytetrahydrofuran-1'-yl)-2-methylfuran-3-carbonyl)hydrazono)-methyl]-2-methylfuran-3-carboxylate* (**7c**). Yield of method A 93%; [a]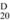 −44.42; IR (KBr): 3,426 (OH), 3,208 (NH), 1,694 (CO-ester), 1,655 (CO-amide), 1,616 cm^−1^ (C=N); ^1^H-NMR (DMSO-d_6_); δ: 1.254 (t, 3H, CH_3(ester)_, *J* = 7.65 Hz), 2.501 (s, 3H, CH_3(furan-1)_), 2.580 (s, 3H, CH_3(furan-2)_), 3.603 (dd, 1H, H-4'b, *J*_3',4'b_ = 2.30 Hz, *J*_4'b,4'a_ = 9.90 Hz), 4.004 (dd, 1H, H-4'a, *J*_3',4'a_ = 4.60 Hz, *J*_4'b,4'a_ = 9.90 Hz), 4.043–4.088 (m, 2H, H-3', H-2'), 4.209 (q, 2H, CH_2(ester)_, *J* = 7.65 Hz), 4.467 (d, 1H, H-1', *J*_1',2'_ = 6.85 Hz), 5.028 (d, 1H, 2'-OH, *J*_2',OH_ = 3.80 Hz), 5.124 (d, 1H, 3'-OH, *J*_3',OH_ = 6.10 Hz), 6.830 (s, 1H, CH_furan-1_), 7.059 (s, 1H, CH_furan-2_), 8.150 (s, 1H, CH=N), 11.409 (s, 1H, NH). After shaking with D_2_O, the NH proton and the two hydroxyl protons disappeared; MS: *m/z* (%), 408 (0.79, M^+^+2), 407 (4.16, M^+^+1), 406 (18.26, M^+^), 211 (100), 154 (32.75); Anal. Calcd for C_19_H_22_N_2_O_8_: C, 56.13; H, 5.47; N, 6.86%; found: C, 56.15; H, 5.46; N, 6.89%.

*N-Benzylidene-5-(2',3'-dihydroxytetrahydrofuran-1'-yl)-2-methylfuran-3-carbohydrazone* (**7d**). Yield of method A 84%; recrystallized from ethanol as colourless needles, mp 86–88 °C; R_f_: 0.345 (chloroform-methanol, 15:1, V/V); [a]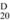 −42.16; IR (KBr): 3,380 (OH and NH), 1,661 (CO-amide), 1,616 cm^−1^ (C=N); ^1^H-NMR (DMSO-d_6_); δ: 2.513 (s, 3H, CH_3(furan)_), 3.608 (dd, 1H, H-4'b, *J*_3',4'b_ = 2.30 Hz, *J*_4'b,4'a_ = 9.95 Hz), 4.012 (dd, 1H, H-4'a, *J*_3',4'a_ = 4.60 Hz, *J*_4'b,4'a_ = 9.95 Hz), 4.065–4.089 (m, 2H, H-3', H-2'), 4.476 (d, 1H, H-1', *J*_1',2'_ = 6.90 Hz), 5.043 (d, 1H, 2'-OH, *J*_2',OH_ = 3.85 Hz), 5.141 (d, 1H, 3'-OH, *J*_3',OH_ = 6.10 Hz), 6.869 (s, 1H, CH_(furan)_), phenyl protons: 7.395–7.436 (m, 3H, *p-*H, *O*-H), 7.672 (d, 2H, *m*-H), 8.340 (s, 1H, CH=N), 11.389 (s, 1H, NH). After addition of D_2_O, the NH proton and the two hydroxyl protons disappeared; MS: *m/z* (%), 331 (2.25, M^+^+1), 330 (10.80, M^+^), 227 (9.18, M^+^-C_6_H_5_CN), 211 (100), 154 (59.63), 77 (9.32, C_6_H_5_); Anal. Calcd for C_17_H_18_N_2_O_5_: C, 61.80; H, 5.50; N, 8.45%; found: C, 61.8; H, 5.49; N, 8.48%.

*5-(2',3'-Dihydroxytetrahydrofuran-1'-yl)-2-methyl-N-(3-phenylallylidene)furan-3-carbohydrazone* (**7e**). Yield of method A 92.5%; recrystallized from ethanol as colourless needles, mp 162–163 °C; R_f_: 0.78 (chloroform:-methanol, 10:1, V/V); [a]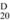 −42.55; IR (KBr): 3,409 (OH), 3,241 (NH), 1,657 (CO-amide), 1,628 cm^−1^ (C=N); ^1^H-NMR (DMSO-d_6_); δ: 2.499 (s, 3H, CH_3(furan)_), 3.598–3.617 (m, 1H, H-4'b), 4.013 (dd, 1H, H-4'a, *J*_3',4'a_ = 3.80 Hz, *J*_4'b,4'a_ = 9.90 Hz), 4.026–4.104 (m, 2H, H-3', H-2'), 4.476 (d, 1H, H-1', *J*_1',2'_ = 6.90 Hz), 5.025 (d, 1H, 2'-OH, *J*_2',OH_ = 3.80 Hz), 5.121 (d, 1H, 3'-OH, *J*_3',OH_ = 6.15 Hz); 6.849 (s, 1H,CH_furan_), 7.00 (bs, 2H, CH=CH), phenyl protons: 7.273–7.302 (m, 1H, *p*-H), 7.355 (t, 2H, *O*-H), 7.585 (d, 2H, *m*-H); 8.124 (d, 1H, CH=N, *J* = 6.15 Hz), 11.264 (s, 1H, NH); MS: *m/z *(%), 358 (0.21, M^+^+2), 357 (0.95, M^+^+1), 356 (3.22, M^+^), 338 (0.11, M^+^-H_2_O), 320 (0.21, M^+^-2H_2_O), 279 (0.52, M^+^-C_6_H_5_), 211 (100), 154 (55.50), 130 (35.56), 77 (8.65, C_6_H_5_), 43 (49, COCH_3_); Anal. Calcd for C_19_H_20_N_2_O_5_: C, 64.03; H, 5.64; N, 7.84%; found: C, 64.04; H, 5.66; N, 7.86%.

#### 3.1.4. Reactions of Compounds **7a,d** with Acetic Anhydride

General Method: A solution of the sugar derivative **7a** or **7d** (1.51 mmol) in a mixture of pyridine (15 mL) and acetic anhydride (15 mL) was kept overnight at room temperature with occasional shaking. Then it was poured onto crushed ice, the di-*O*-acetyl derivative that separated out, was filtered off, washed with water, and dried.

*1'-[2-Methyl-3-(2-((2-phenyl-2H-1,2,3-triazol-4-yl)methylene)hydrazinecarbonyl)furan-5-yl]-tetra-hydrofuran-2',3'-diyl diacetate* (**8a**). Yield 76.5%; recrystallized from dilute ethanol as colourless needles, mp 179–181 °C; R_f_ 0.5 (hexane-ethyl acetate, 2:1, V/V); [a]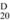 −47.75; IR (KBr): 3,217 (NH), 1,754 (OAc), 1,647 (CO-amide), 1,601 cm^−1^ (C=N); ^1^H-NMR (CDCl_3_); δ: 2.061, 2.139 (2s, 6H, 2OAc), 2.677 (s, 3H, CH_3(furan)_), 3.984–3.968 (m, 1H, H-4'b), 4.371 (dd, 1H, H-4'a, J_3',4'a_ = 4.60 Hz, J_4'b,4'a_ = 9.95 Hz), 4.925 (d, 1H, H-1', J_1',2'_ = 6.10 Hz), 5.492–5.532 (m, 2H, H-3', H-2'), 6.744 (bs, 1H, CH_furan_), 7.462–7.478 (m, 1H, p-H), 7.531–7.625 (m, 2H, O-H), 8.019–8.141 (m, 4H, m-H, CH=N, CH_triazole_), and 12.239 (s, 1H, NH); MS: m/z (%), 482 (0.61, M^+^+1), 481 (1.97, M^+^), 363 (22.58), 362 (100), 295 (67.47), 137 (21.53), 115 (19.79), 77 (15.78); Anal. Calcd for C_23_H_23_N_5_O_7_: C, 57.35; H, 4.82; N, 14.53%; found: C, 57.38; H, 4.82; N, 14.55%.

*1'-[3-(2-Benzylidenehydrazinecarbonyl)-2-methylfuran-5-yl]tetrahydrofuran-2',3'-diyl diacetate *(**8d**). Yield 80%; recrystallized from dilute methanol as colourless needles, mp 81–82 °C; R_f_ 0.5 (hexane-ethyl acetate, 2:1, V/V); [a]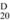 −48.37; IR (KBr): 3,248 (NH), 1,752 (OAc), 1,654 (CO-amide), 1,582 cm^−1^ (C=N); ^1^H-NMR (CDCl_3_); δ: 2.070, 2.125 (2s, 6H, 2OAc), 2.602 (s, 3H, CH_3(furan)_), 3.954–3.974 (m, 1H, H-4'b), 4.381–4.350 (m, 1H, H-4'a), 4.950 (bs, 1H, H-1'), 5.417–5.524 (m, 2H, H-3', H-2'), 6.620 (s, 1H, CH_furan_), 7.519–7.549 (m, 5H, phenyl protons), 7.883 (d, 1H, CH=N, *J* = 7.65 Hz), 9.550 (s, 1H, NH); MS: *m/z* (%), 415 (0.39, M^+^+1), 414 (0.81, M^+^), 295 (100), 77 (2.61); Anal. Calcd for C_21_H_22_N_2_O_7_: C, 60.86; H, 5.37; N, 6.77%; found: C, 60.86; H, 5.35; N, 6.76%.

*Ethyl 5-(3,5-dihydroxy-1,4-dioxan-2-yl)-2-methylfuran-3-carboxylate* (**9**). A solution of **2b,4b** mixture (12.96 g, 50.62 mmol) in distilled water (20 mL) was treated dropwise with a solution of sodium metaperiodate (10.825 g, 50.62 mmol) in distilled water (20 mL) with continuous stirring for 3 h. The dialdehyde that separated out was filtered off, washed with little water, and dried, yield 67%; R_f_: 0.38 (chloroform-methanol, 15:1, V/V). Recrystallized from ethanol as colourless needles, mp 111–113 °C (Lit. [22], 111–113 °C); IR (KBr): 3,426 (OH), 1,712 cm^−1^ (CO-ester); ^1^H-NMR (CDCl_3_); δ: 1.293–1.326 (m, 3H, CH_3(ester)_), 2.519 (s, 3H, CH_3(furan)_), 3.380–3.428 (m, 1H, H-6b), 3.745–3.947 (m, 1H, H-6a), 4.222–4.387 (m, 2H, 3-OH, 5-OH), 4.275 (q, 2H, CH_2(ester)_), 4.609–4.745 (m, 1H, H-5), 5.112–5.125 (m, 1H, H-3), 5.514–5.534 (m, 1H, H-2), and 6.677 (s, 1H, CH_furan_).

#### 3.1.5. Reactions of **9** with a Number of Aroylhydrazines

General Method: A solution of **9** (2.0 g, 7.353 mmol) in ethanol (10 mL) containing acetic acid (0.1 mL) was treated with the corresponding aroylhydrazine (14.71 mmol) in ethanol (10 mL). The reaction mixture was refluxed on water bath for 15 min, the bisaroylhydrazone derivative that separated out was filtered off, washed with little ethanol, and dried.

*Ethyl 5-[2-(2-benzoylhydrazono)-1-(2-(2-benzoylhydrazono)ethoxy)ethyl]-2-methylfuran-3-carboxylate *(**10a**). Yield 61%; recrystallized from ethanol-chloroform as colourless needles, mp 204–206° C; R_f_: 0.55 (chloroform-methanol, 10:1, V/V); IR (KBr): 3,280, 3,197 (2NH), 1,715 (CO-ester), 1,658 (CO-amide), 1,591 cm^−1^ (C=N); ^1^H-NMR (DMSO-d_6_); δ: 1.223 (t, 3H, CH_3(ester)_, *J* = 6.90 Hz ), 2.524 (s, 3H, CH_3(furan)_), 4.086–4.199 (m, 4H, CH_2_, CH_2(ester)_), 5.150 (d, 1H, CH, *J* = 6.85 Hz), 6.736 (s, 1H, CH_furan_), phenyl protons: 7.472–7.476 (m, 4H, *O*-H), 7.550–7.546 (m, 2H, *p*-H), 7.794–7.837 (m, 4H, *m-*H), 7.719 (bs, 1H, CH=N_1_), 7.912 (d, 1H, CH=N_2_, *J* = 6.10 Hz), 11.673 (s, 1H, NH_3_), 11.777 (s, 1H, NH_4_); MS: *m/z* (%), 472 (0.57, M^+^-H_2_O), 354 (56.88), 181 (31.16), 173 (29.44), 153 (23.60), 147 (93.31), 121 (41.45), 106 (49.34), 105 (100), 104 (23.78), 78 (21.08), 77 (99.13), 51 (77.47), 50 (24.02); Anal. Calcd for C_26_H_26_N_4_O_6_: C, 63.65; H, 5.35; N, 11.43%; found: C, 63.66; H, 5.34; N, 11.42%.

*Ethyl 5-[2-(2-(p-toluoyl)hydrazono)-1-(2-(2-(p-toluoyl)hydrazono)ethoxy)ethyl]-2-methylfuran-3-carboxylate* (**10b**). Yield 58%; recrystallized from ethanol-chloroform as colourless needles, mp 204–206 °C; R_f_: 0.22 (chloroform-methanol, 15:1, V/V); IR (KBr): 3,305, 3,183 (2NH), 1,717 (CO-ester), 1,651 (CO-amide), 1,625, 1,612 cm^−1^ (C=N); ^1^H-NMR (DMSO-d_6_); δ: 1.222 (t, 3H, CH_3(ester)_, *J* = 6.85 Hz), 2.327 (s, 6H, 2CH_3(toluoyl)_), 2.522 (s, 3H, CH_3(furan)_), 4.103–4.196 (m, 4H, CH_2_, CH_2(ester)_), 5.132 (d, 1H, CH, *J* = 6.90 Hz), 6.727 (s, 1H, CH_furan_), aromatic protons: 7.265–7.280 (m, 4H, *O*-H), 7.708–7.747(m, 5H, *m*-H, CH=N_1_), 7.899 (d, 1H, CH=N_2_, *J* = 6.10 Hz), 11.601 (s, 1H, NH_3_), 11.716 (s, 1H, NH_4_); MS: *m/z* (%), 518 (0.09, M^+^), 500 (0.18, M^+^-H_2_O), 368 (31.86), 161 (44.53), 161 (44.53), 119 (100), 91 (50.53), 65 (19.49); Anal. Calcd for C_28_H_30_N_4_O_6_: C, 64.87; H, 5.85; N, 10.78%; found: C, 64.85; H, 5.83; N, 10.80%.

*Ethyl 5-[2-(2-(p-anisoyl)hydrazono)-1-(2-(2-(p-anisoyl)hydrazono)ethoxy)ethyl]-2-methylfuran-3-carboxylate* (**10c**). Yield 57%; recrystallized from ethanol-chloroform as colourless needles, mp 204–206 °C; R_f_: 0.32 (chloroform:-methanol, 10:1, V/V); IR (KBr): 3,268, 3,212 (2NH), 1,718 (CO-ester), 1,645 (CO-amide), 1,610 cm^−1^ (C=N); ^1^H-NMR (DMSO-d_6_); δ: 1.225 (t, 3H, CH_3(ester)_, *J* = 6.15 Hz), 2.525 (s, 3H, CH_3(furan)_), 3.776 (s, 6H, 2OCH_3_), 4.156–4.199 (m, 4H, CH_2_, CH_2(ester)_), 5.127 (d, 1H, CH, *J* = 6.15 Hz), 6.726 (s, 1H, CH_furan_), aromatic protons: 6.700–7.008 (m, 4H, *m*-H), 7.799–7.837 (m, 4H, *O*-H), 7.707 (bs, 1H, CH=N_1_), 7.895 (d, 1H, CH=N_2_, *J* = 6.10 Hz), 11.554 (s, 1H, NH_3_), 11.653 (s, 1H, NH_4_); MS: *m/z* (%), 532 (0.15, M^+^-H_2_O), 135 (100); Anal. Calcd for C_28_H_30_N_4_O_8_: C, 61.05; H, 5.47; N, 10.19%; found: C, 61.08; H, 5.49; N, 10.18%.

*Ethyl 5-[2-(2-(p-chlorobenzoyl)hydrazono)-1-(2-(2-(p-chlorobenzoyl)hydrazono)ethoxy)ethyl]-2-methylfuran-3-carboxylate *(**10d**). Yield 62%; recrystallized from ethanol-chloroform as colourless needles, mp 204–206 °C; Rf: 0.4 (chloroform-methanol, 15:1, V/V); IR (KBr): 3,270, 3,215 (2NH), 1,719 (CO-ester), 1,650 (CO-amide), 1,612 cm^−1^ (C=N); Anal. Calcd for C_26_H_24_Cl_2_N_4_O_6_: C, 55.80; H, 4.33; N, 10.02; Cl, 12.69%; found: C, 55.82; H, 4.32; N, 10.02; Cl, 12.68%.

#### 3.1.6. Reactions of Compounds **10a–d** with Yellow Mercuric Oxide

General Method: A solution of **10a–d** (4.5 mmol) in dry ether (50 mL) was stirred with yellow mercuric oxide (3.0 g), magnesium oxide (0.3 g), and iodine (2.5 g) at room temperature for 48 h under anhydrous condition. The reaction mixture was filtered off, and the filtrate washed with potassium iodide solution, sodium thiosulphate, and water, respectively, then dried over anhydrous sodium sulphate. On evaporation of the dried filtrate a yellow syrup was obtained, which was crystallized from ethanol. An additional crop was obtained by extracting the inorganic residue with chloroform which upon concentration and dilution with ethanol yielded the same product.

*Ethyl 2-methyl-5-(5-phenyl-1,3,4-oxadiazole-2-carbonyl)furan-3-carboxylate* (**11a**). Yield 36%; recrystallized from ethanol as colourless needles, mp 168–170 °C; R_f_: 0.41 (hexane-ethyl acetate, 6:1, V/V); IR (KBr): 1,715 (CO-ester), 1,660 (C=O), 1,594 cm^−1^ (C=N); ^1^H-NMR (CDCl_3_); δ: 1.391 (t, 3H, CH_3(ester)_, *J* = 6.85 Hz), 2.778 (s, 3H, CH_3(furan)_), 4.344 (q, 2H, CH_2(ester)_, *J* = 6.85 Hz), phenyl protons: 7.565 (t, 2H, *O*-H), 7.611-7.637 (m, 1H, *p*-H), 8.210-8.224 (m, 2H, *m*-H), 8.491 (s, 1H, CH_furan_); MS: *m/z* (%), 328 (3.07, M^+^+2), 327 (20.48, M^+^+1), 326 (100, M^+^), 181 (22.15), 153 (32.73), 145 (61.44), 77 (49.04); Anal. Calcd for C_17_H_14_N_2_O_5_: C, 62.57; H, 4.32; N, 8.59%; found: C, 62.57; H, 4.33; N, 8.60%.

*Ethyl 2-methyl-5-(5-(p-tolyl)-1,3,4-oxadiazole-2-carbonyl)furan-3-carboxylate* (**11b**). Yield 39%; recrystallized from ethanol as colourless needles, mp 185–187 °C; R_f_: 0.52 (hexane-ethyl acetate, 6:1, V/V); IR (KBr): 1,715 (CO-ester), 1,660 (C=O), 1,611 cm^−1^ (C=N); ^1^H-NMR (CDCl_3_); δ: 1.390 (t, 3H, CH_3(ester)_, *J* = 6.90 Hz), 2.457 (s, 3H, CH_3(tolyl)_), 2.775 (s, 3H, CH_3(furan)_), 4.348 (q, 2H, CH_2(ester)_, *J* = 6.90 Hz), phenyl protons: 7.361 (d, 2H, *O*-H), 8.098 (d, 2H, *m*-H), 8.482 (s, 1H, CH_furan_); Anal. Calcd for C_18_H_16_N_2_O_5_: C, 63.53; H, 4.76; N, 8.25%; found: C, 63.52; H, 4.74; N, 8.23%.

*Ethyl 2-methyl-5-*[5-(p-anisyl)-1,3,4-oxadiazole-2-carbonyl]*furan-3-carboxylate* (**11c**). Yield 21%; recrystallized from ethanol as colourless needles, mp 182–183 °C; R_f_: 0.41 (hexane-ethyl acetate, 6:1, V/V); IR (KBr): 1,714 (CO-ester), 1,657 (C=O), 1,606 cm^−1^ (C=N); Anal. Calcd for C_18_H_16_N_2_O_6_: C, 60.66; H, 4.55; N, 7.86%; found: C, 60.67; H, 4.53; N, 7.86%.

*Ethyl 2-methyl-5-*[5-(p-chlorophenyl)-1,3,4-oxadiazole-2-carbonyl]*furan-3-carboxylate* (**11d**). Yield 39%; recrystallized from ethanol as colourless needles, mp 177–178 °C; R_f_: 0.59 (hexane-ethyl acetate, 6:1, V/V); IR (KBr): 1,723 (CO-ester), 1,667 (C=O), and 1,600 cm^−1^ (C=N); ^1^H-NMR (CDCl_3_); δ: 1.391 (t, 3H, CH_3(ester)_, *J* = 6.85 Hz), 2.778 (s, 3H, CH_3(furan)_), 4.353 (q, 2H, CH_2(ester)_, *J* = 6.85 Hz), *p*-chlorophenyl protons: 7.550 (d, 2H, *m*-H), 8.158 (d, 2H, *O*-H), 8.482 (s, 1H, CH_furan_); ^1^H-NMR (DMSO-d_6_); δ: 1.293 (t, 3H, CH_3(ester)_, *J* = 6.85 Hz), 2.692 (s, 3H, CH_3(furan)_), 4.269 (q, 2H, CH_2(ester)_, *J* = 6.85 Hz), *p*-chlorophenyl protons: 7.700 (d, 2H, *m*-H), 8.082 (d, 2H,*O*-H), 8.170 (s, 1H, CH_furan_); MS: *m/z* (%), 362/360 (100, 42, M^+^), 181 (78.7), 179 (71.29), 153 (78.02), 141/139 (11.12, 33.56), 137 (39.6), 113/111 (10.89, 33.86); Anal. Calcd for C_17_H_13_ClN_2_O_5_: C, 56.62; H, 3.64; N, 7.75; Cl, 9.81%; found: C, 56.60; H, 3.63; N, 7.77; Cl, 9.83%.

### 3.2. Pharmacological Screening

#### 3.2.1. MAO-B Activity

##### 3.2.1.1. Enzyme Preparation

Rabbit brain was homogenized in 9 volumes of ice-cold 0.1 M sodium phosphate buffer (pH 7.4) with an Ultra-Turrax 18/2 homogenizer. The separated mitochondrial fraction was suspended in phosphate buffer to give a final volume of 1 mL g^−1^ weight of tissue [40].

##### 3.2.1.2. Enzyme Assay

Monoamine oxidase-B activity was assayed in presence and absence of the examined compounds each separately using benzylamine as substrate by continuous recording on a Pye Unicam SP8-100 Double Beam Spectrophotometer of the increase in extinction at 250 nm produced at 38 °C.

##### 3.2.1.3. Determination of V_max_ and K_m_

The V_max_ and K_m_ of MAO-B catalyzed reaction in presence or absence of each examined compound 7c and 7e was carried out by plotting the rate of the reaction (V) against substrate concentration [S], each separately.

#### 3.2.2. Antibacterial and Antifungal Screening

The antibacterial and antifungal activities of synthesized compounds **3b**, **6**, **7c,e**, **10a–c** and **11a,b,d** against four bacterial species (*Escherichia coli*, *Bacillus* sp., *Staphylococcus* sp., and *Sarcina* sp.) and six fungal species (*Aspergillus niger*, *Aspergillus fmigatus*, *Alternaria* sp., *Fusarium* sp., *Chaetomium* sp., and *Penicillium* sp.). have been studied by using the Nutrient Agar (NA) and Sabouraud Dextrose Agar (SDA) diffusion methods, respectively in DMSO solvent. The bacteria were subcultured on Nutrient Agar medium (NA), whereas, fungi were subcultured on Sabouraud Dextrose Agar (SDA). The composition is given in g/L unless otherwise stated. The pH value of the media was adjusted to 7 ± 0.1 prior to sterilization with 0.1 M sodium hydroxide or hydrochloric acid. All media were prepared with distilled water and sterilized by autoclaving at 121 °C for 20 min. *Nutrient Agar (NB):* Peptone, 5; beef extract, 3; NaCl, 5; agar, 20. *Sabouraud Dextrose Agar (SDA)*: Peptone, 10; glucose, 40; agar, 20. The stock solutions (1 mg/mL) of the test chemicals were prepared by dissolving 10 mg of the test compounds in 10 mL dimethylsulphoxide (DMSO) solvent. Petri plates (150 mm × 15 mm) were prepared by pouring 60 mL of SDA and allowed to solidify. Plates were dried and 1 mL of each standardized inoculums suspension was poured and uniformly spread. The excess inoculums was drained and the inoculums was allowed to dry for 15 min. Eight equidistant wells were made in the medium using a sterile cork borer (6 mm in diameter and 50 μL of the test chemicals (1 mg/mL) diluted in DMSO 2% were placed into the wells. The Petri-dishes containing bacterial and fungi species were incubated at 37 °C for 24 h, and 48 h, respectively. The tests were carried in triplicate. The antimicrobial activity was measured as the diameter (mm) of clear zone of growth inhibition.

## 4. Conclusions

Some new *C*-glycoside derivatives and heterocyclic derivatives of carbohydrate have been prepared and their physical and biological properties were studied. It was found that compounds with highly conjugated systems are acting as MAO-B activators, as well as antibacterial agents, meanwhile all tested compounds have antifungal activities.
